# Use of JAK Inhibitors in Lichen Planus: An Update

**DOI:** 10.3390/medicina61061056

**Published:** 2025-06-08

**Authors:** Dario Didona, Raffaele Dante Caposiena Caro, Laura Calabrese, Martina D’Onghia, Giulia Galluccio, Matteo Riccardo Di Nicola, Alessandra Rallo, Giovanni Paolino

**Affiliations:** 1 Rare Skin Diseases Center, Istituto Dermopatico dell’Immacolata, Istituto Dermopatico dell’Immacolata-IRCCS, 00167 Rome, Italy; a.rallo@idi.it; 2 Dermatologic Clinic, Hospital Maggiore of Trieste, University of Trieste, 34127 Trieste, Italy; raffaele.caposienacaro@asugi.sanita.fvg.it; 3 Dermatology Unit, Department of Medical, Surgical and Neurological Sciences, University of Siena, 53100 Siena, Italy; laura.calabrese@unisi.it (L.C.); martina.donghia@gmail.com (M.D.); giulia.galluccio95@gmail.com (G.G.); 4 Institute of Dermatology, Catholic University of the Sacred Heart, 00168 Rome, Italy; 5 Istituto Zooprofilattico Sperimentale del Piemonte, Liguria e Valle d’Aosta, 10154 Turin, Italy; matteoriccardo.dinicola@izsplv.it; 6 Dermatology Unit, Department of Clinical Internal, Anesthesiological and Cardiovascular Science, University of La Sapienza, 00185 Rome, Italy; 7 Unit of Dermatology and Cosmetology, IRCCS Ospedale San Raffaele, 20132 Milan, Italy; paolino.giovanni@hsr.it; 8 Faculty of Medicine and Surgery, Vita-Salute San Raffaele University, 20132 Milan, Italy

**Keywords:** cutaneous lichen planus, Janus kinase inhibitors, lichen planus, oral lichen planus, therapy

## Abstract

Lichen planus (LP) is a chronic inflammatory disorder affecting approximately 1% of the population. It presents with a wide range of clinical manifestations, mainly involving the skin, mucosal surfaces, and skin appendages, and is often characterized by a relapsing course and variable response to treatment. Although several therapeutic strategies are available, many are off-label and show limited efficacy in resistant forms. Increasing evidence points to the central role of the JAK/STAT signaling pathway in the immunopathogenesis of LP, with cytokines such as interferon-gamma and interleukin-21 playing key roles in sustaining chronic inflammation. Based on this rationale, Janus kinase (JAK) inhibitors have recently been proposed as potential therapeutic agents in LP. This review explores the biological basis for their use and systematically summarizes the existing clinical evidence on the use of JAK inhibitors in cutaneous, mucosal, appendageal, and nail variants of LP. The preliminary data suggests favorable outcomes in many patients with difficult-to-treat disease, with an acceptable safety profile. Further prospective trials are needed to establish their definitive role in the management of LP.

## 1. Introduction

**Figure 1:**
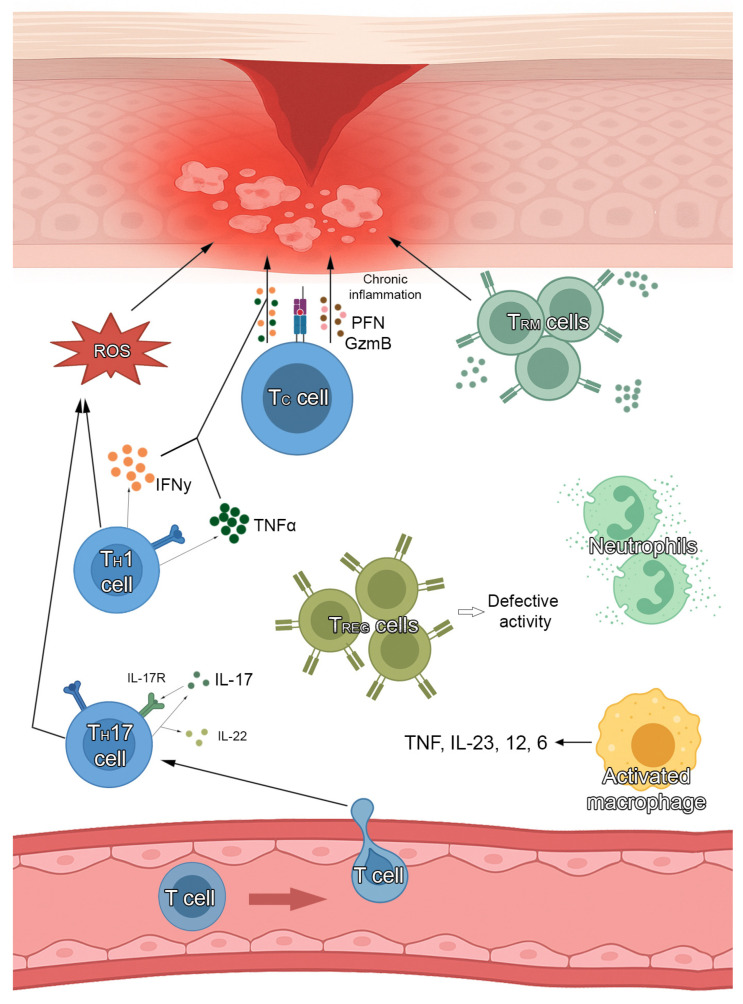
Pathogenesis of lichen planus.

Lichen planus (LP) is a chronic inflammatory disorder characterized by a dense infiltration of inflammatory T cells arranged in a band-like pattern in the upper dermis [1]. LP can affect the skin, mucous membranes (oral mucosa, genital area and conjunctiva), appendages (hair and nails) and esophagus [1]. Cutaneous LP (CLP) predominantly affects middle-aged adults, with an estimated incidence ranging between 0.14% and 1.27% with no sex or racial preferences [2], while mucosal LP has a prevalence of 0.89%, showing an higher incidence in South America and a more frequent involvement of female patients [3]. The pathogenesis of LP is complex and involves a T cell-mediated immune response and a combination of genetic susceptibility and environmental triggers, such as stress, infections, and alterations of the mucosal microbiome (Figure 1) [4]. Indeed, CD8+ cytotoxic T lymphocytes play a pivotal role in the destruction of basal keratinocytes, contributing to the characteristic interface dermatitis observed in histopathological examinations, while cytokine dysregulation, including increased expression of interferon-gamma (IFN-γ) and tumor necrosis factor-alpha (TNF-α), further amplifies the inflammatory cascade, leading to tissue damage [4]. Despite the significant advances in understanding the immunopathogenesis of LP, current treatment options can be suboptimal in refractory cases. In addition, conventional therapies such as corticosteroids, calcineurin inhibitors, and systemic immunosuppressants, like azathioprine or mycophenolate mofetil, can be associated with several adverse effects, indicating the need for new therapeutic approaches. 

Janus kinase (JAK) inhibitors have emerged as a promising therapeutic option for several skin diseases, such as psoriasis, vitiligo, atopic dermatitis, and alopecia areata [5]. By targeting the JAK/signal transducer and activators of transcription (STAT) signaling pathway, these agents can potentially reduce the aberrant T cell activation and cytokine production implicated in LP development [5]. Preliminary evidence suggests that JAK inhibitors (JAKI) may offer an effective alternative for patients with recalcitrant LP, providing a target and safer approach compared to traditional immunosuppressive therapies [6]. The aim of our review was to explore the rationale for the use of JAKI as a novel therapeutic frontier to achieve better disease control and improve patient outcomes.

## 2. Clinical Features of Cutaneous Lichen Planus

**Figure 2:**
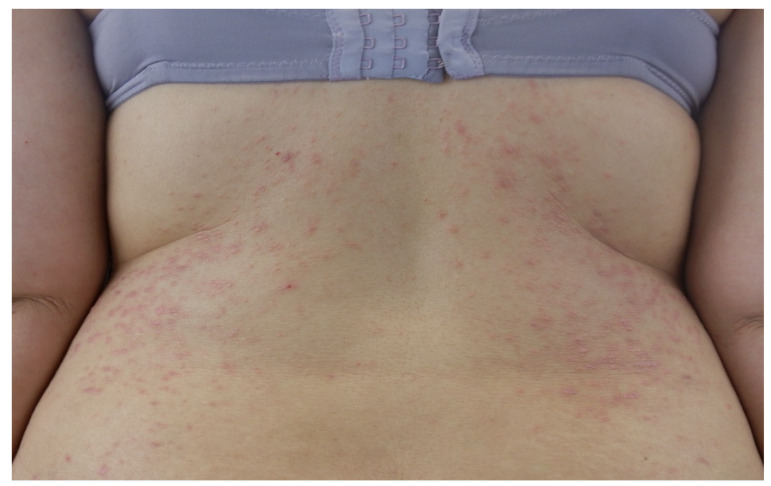
Symmetric, polygonal, flat, purplish papules on the back of a young woman with cutaneous lichen planus.

CLP classically presents as a papular eruption, characterized by pruritic, polygonal, flat-topped violaceous papules, typically distributed symmetrically over the flexor surfaces of the extremities (Figure 2) [1]. Papules are usually a few millimeters in diameter, but may coalesce to form larger plaques [1]. Dermoscopy often reveals highly characteristic reticular white lines, known as Wickham’s striae [7]. Asymptomatic CLP is extremely rare because CLP is associated with intense pruritus [1]. In addition, the development of new papules on areas of cutaneous injury (Köbner’s phenomenon) is a common characteristic of CLP and it is usually induced by scratching [1]. While the classic form of CLP is the most prevalent, other possible variants can be observed because the polygonal papules can be replaced by several different lesions, including hyperkeratotic nodules, atrophic papules, and hyperpigmented patches [1,8]. Hypertrophic LP, also known as verrucous LP, is characterized by brown to purple-gray hyperkeratotic nodules and plaques that commonly involve symmetrically the anterior legs and the interphalangeal joints [9].

**Figure 3:**
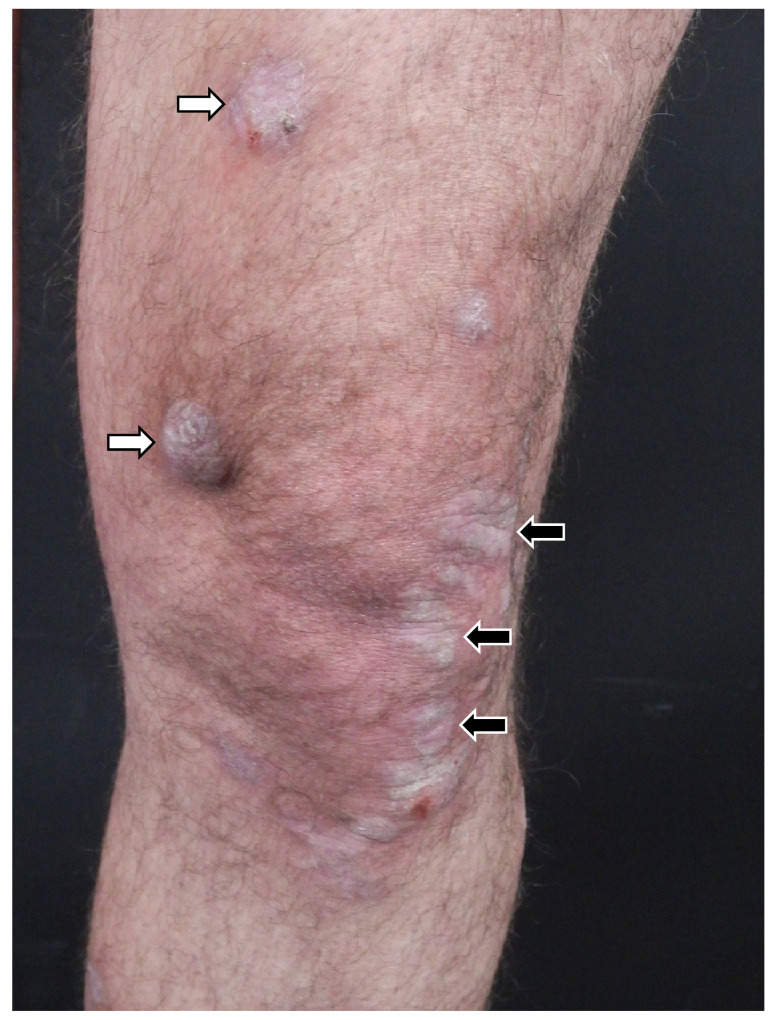
Coexistence of hypertrophic lichen (white arrows) and atrophic lichen (black arrows) in a male patient.

**Figure 4:**
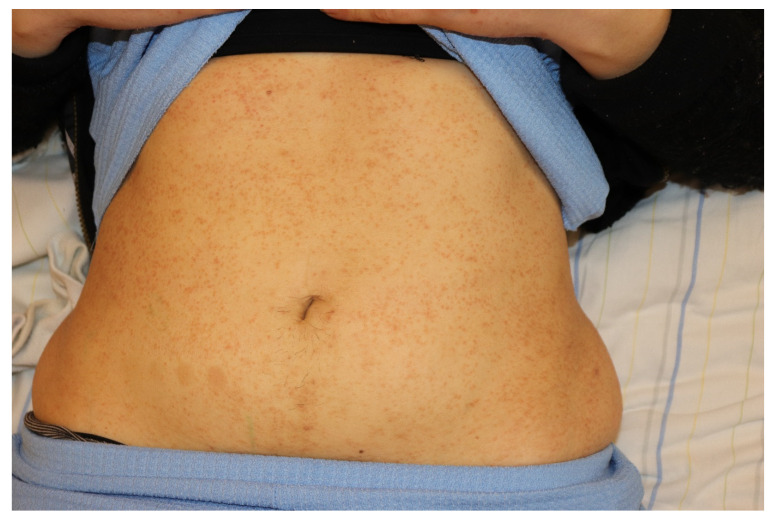
Multiple, pinhead-like, erythematous papules in a patient with exanthematous lichen planus.

The occasional development of cutaneous squamous cell carcinoma has been reported in patients with longstanding LP [10]. Atrophic LP is a rare variant that can develop in areas previously affected by other forms of LP (Figure 3, black arrows) [8]. Atrophic LP is characterized by well-demarcated white-bluish or brown papules and plaques, typically appearing after the resolution of annular or ulcerative lesions [11]. Commonly affected sites include the axillae, glans penis, lower extremities, and trunk [11]. LP pigmentosus is characterized by dark brown to gray macular patches on sun-exposed areas and it is usually described in patients with phototype III and IV according to Fitzpatrick’s skin phototype scale [12]. Inverse LP is a variant that primarily affects intertriginous areas, characterized by erythematous patches with poorly defined borders and hyperkeratosis [8]. CLP is often a localized manifestation, but less frequently a generalized eruption may develop [1]. In this eruptive variant, also known as exanthematous LP, multiple concomitant eruptive lesions may display different morphologies, suggesting a chronological evolution of individual papules and macules (Figure 4) [13].

In the vesiculobullous subtype, patients develop vesicles and blisters over typical lesions of LP, especially on the lower extremities [1]. Actinic LP is a rare subtype presenting as nummular patches or plaques with a typical hypopigmented halo surrounding a hyperpigmented center. This variant involves sun-exposed areas of pigmented skin, including the face, which is usually spared in other subtypes of CLP [1]. Actinic LP seems to be triggered by UV light and is more prevalent in Middle Eastern countries [1]. In contrast with classical LP, features like pruritus and Köbner’s phenomenon are not usually reported [1]. Annular LP usually involves the male genitalia, axilla and groins, and it is characterized by a ring shape that may arise from either the coalescence of multiple lichenoid papules into a circular configuration or the expansion of a papule or plaque with central regression [1]. Linear LP is a rare subtype that affects fewer than 1% of all patients with LP, with a higher incidence in Japan [1,2]. In this form, itchy papules develop in a linear way, usually unilateral, following a blaschkoid (blaschkoid LP) or, less frequently, a dermatomal distribution (zosteriform LP) [1,2]. This peculiar linear arrangement should be distinguished from a linear pattern of trauma due to Köbner’s phenomenon [1,2]. Inverse LP is a variant that primarily affects intertriginous areas, such as the axillae, inguinal folds, gluteal cleft, flexural surfaces of the limbs, and submammary folds [1,2]. In these areas, lesions may deviate from the classic LP morphology, appearing as erythematous patches with poorly defined borders and lichenification [1,2].

## 3. Clinical Features of Oral Lichen Planus

**Figure 5:**
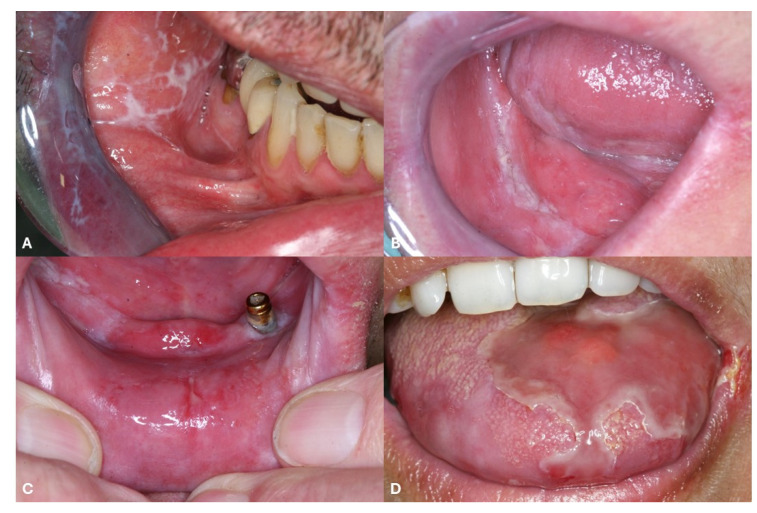
(**A**) White stripes of the oral mucosa in a patient with reticular oral lichen planus. (**B**) White plaques of the oral mucosa in a patient with non-erosive, plaque-type oral lichen planus. (**C**) Erosions of the lower gingiva in a male patient with erosive oral lichen planus. (**D**) Massive ulceration in a female patient with ulcerative oral lichen planus.

Oral lichen planus (OLP) is the most frequent type of mucosal LP [1,14]. OLP can be isolated but often arises concomitantly with CLP [1,14]. Indeed, about 15% of patients with OLP are also affected by CLP [1,14]. The clinical presentation of OLP involves a range from asymptomatic white keratotic lesions (non-erosive OLP) to painful erosions and ulcerations (erosive OLP) [1,14]. Non-erosive OLP includes reticular OLP and plaque OLP [1,14]. Reticular OLP (Figure 5A) is characterized by asymptomatic, reticular white lesions that form a lace-like pattern and most commonly affects the buccal mucosa symmetrically, while plaque OLP is more frequent prevalent in tobacco smokers and presents with large and homogenous white patches and plaque-like keratotic lesions (Figure 5B) [1,14]. Erosive OLP shows painful erosions (Figure 5C) or ulcerations (Figure 5D), often associated with Wickham’s striae, that should be differentiated from pemphigus vulgaris (PV) lesions [15]. Indeed, it has been reported that erosive OLP can be associated with IgG serum antibodies against desmoglein 3, one of the characteristic target of PV [16,17]. Regular screening for oral cancer in OLP is recommended. Indeed, Fitzpatrick et al. reported that 85 (1.09%) of 7806 OLP patients and 4 (3.2%) of 125 patients with oral lichenoid lesions developed oral squamous cell carcinoma [18]. Furthermore, Georgakopoulou et al. reported a malignant transformation rate of 12.5% [19].

## 4. Clinical Features of Lichen Planus of Appendages

**Figure 6:**
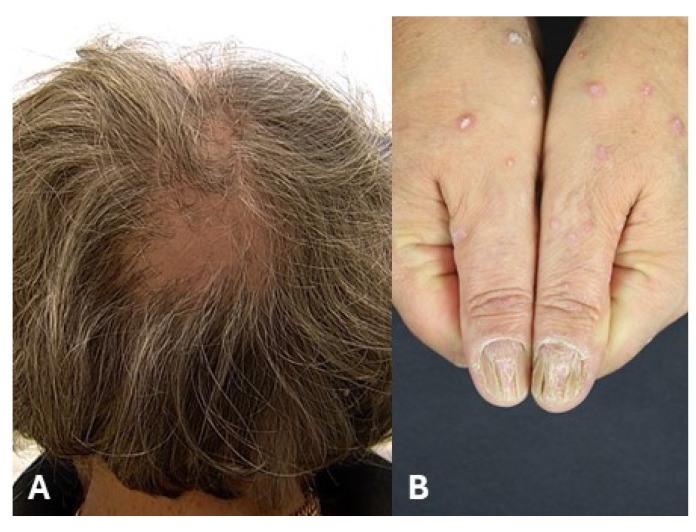
(**A**) Cicatricial alopecia in a woman with lichen planopilaris. (**B**) Destruction of the architecture of the nail in a female patient with nail lichen planus.

Lichen planopilaris (LPP) is a lymphocytic scarring alopecia characterized by irregular patchy hair loss [20,21]. LPP predominantly affects female patients (M:F ratio = 1:2) between 30 and 75 years of age [20,21]. Three major subclinical variants have been described: Classic LPP (Figure 6A), frontal fibrosing alopecia (FFA) and Graham–Little–Piccardi–Lasseur’s syndrome. FFA is one of the most characteristic variants of LPP, showing a typical receding frontotemporal hairline [20,21]. FFA mostly occurs in postmenopausal women and may also affect the eyebrows [20,21]. Finally, Graham–Little–Piccardi–Lasseur’s syndrome shows multifocal scarring alopecia of the scalp associated with non-scarring alopecia of the groin and axillar region as well as hyperkeratotic plaques on the trunk [22]. Nail lichen planus (NLP) occurs in approximately 15% of patients with CLP and may present as an isolated nail disorder [23,24]. The diagnosis is primarily based on a history and clinical examination [23,24]. NLP affects the nail matrix, manifesting as longitudinal ridging/splitting and nail plate thinning, whereas nail bed involvement is characterized by onycholysis (Figure 6B) and nail bed hyperkeratosis [23,24]. Irreversible changes include anonychia, nail plate atrophy, and pterygium [23,24].

## 5. JAK Inhibitors and Their Rationale for the Treatment of Lichen Planus

**Figure 7:**
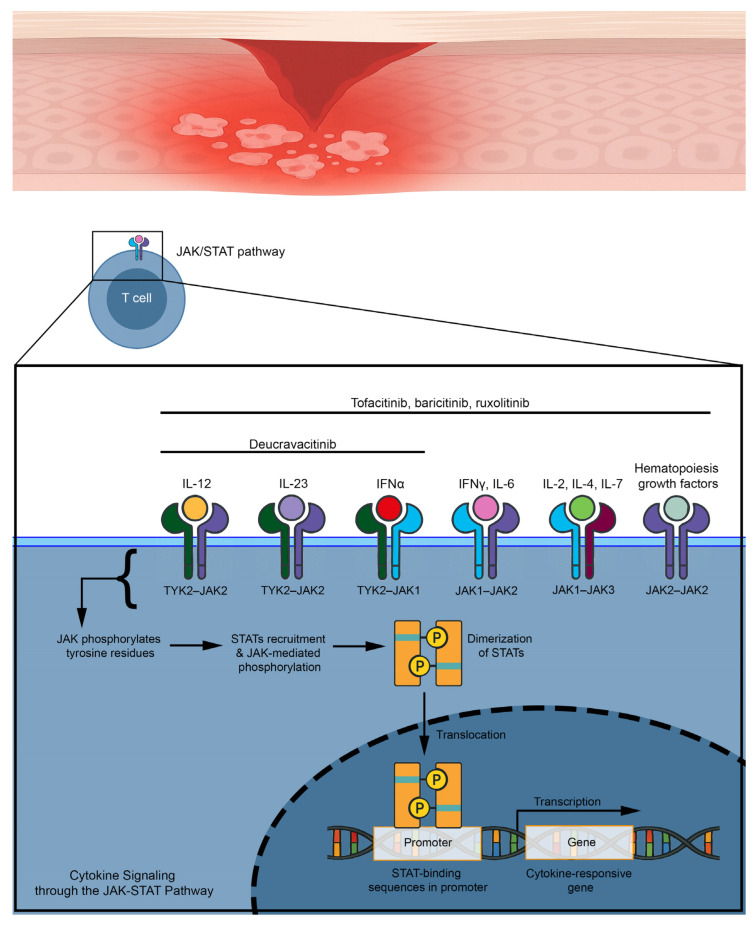
Mechanism of action of JAK inhibitors in lichen planus.

The primary function of protein kinases is to transfer phosphate groups from adenosine triphosphate or guanosine triphosphate to the hydroxyl groups of amino acids of their protein targets (Figure 7) [25]. This mechanism is also important for cytokine receptors, which do not have an intrinsic enzymatic activity to initiate an inflammatory cascade [26]. A large number of cytokines, such as interleukin (IL) 21 and IL-23 and interferons such as IFN-γ, interact with type I and II cytokine receptors, which strongly rely on JAK for signal transduction because of their lack of intrinsic enzyme activity [26]. After binding, recruited JAK starts a signaling cascade from the cellular membrane to the nucleus: Type I and II cytokine receptors undergo oligomerization and recruitment of JAK, which phosphorylates tyrosine residues and activates STAT proteins, triggering them to undergo dimerization and translocate into the nucleus, leading to a modulation of gene expression [27]. Several studies support a role of the JAK–STAT pathway in LP pathogenesis [28,29]. Indeed, Pietschke et al. found out that IL-21 is highly expressed in cutaneous LP [28]. IL-21 stimulates the differentiation and function of CD4+ T cells, CD8+ T cells, and NK cells, which are all present in lesional skin in patients with LP [30,31] and exerts its intracellular effects through a STAT1/STAT3-dependent mechanism that activates STAT1 [29]. Furthermore, Shao et al. demonstrated that IFN-γ enhances cell-mediated cytotoxicity against keratinocytes via JAK2/STAT1 in LP [29]. Furthermore, JAK has been shown to have a role in modulating the IFN-γ/C-X-C motif chemokine ligand 10 (CXCL10) axis implicated in NLP pathogenesis [32]. Accordingly, therapeutic targeting of this pathway has been used with success in all LP forms [32].

## 6. Use of JAK Inhibitors for Cutaneous Lichen Planus

Oral tofacitinib was initially reported as effective in hypertrophic LP. Indeed, Seiringer et al. described the case of a 51-year-old male patient with a 30-year history of verrucous plaques who was unresponsive to several systemic therapies, including apremilast, ixekizumab, and guselkumab [33]. After starting oral tofacitinib 5 mg twice per day, his NRS pruritus dropped from 10 to zero within 20 weeks and the patient showed a massive improvement of the clinical picture [33]. Furthermore, Youssef et al. described another case of refractory hypertrophic LP that completely resolved within three months after starting oral tofacitinib 10 mg twice daily [34]. Upadacitinib was also successfully used in a 46-year-old woman with 8 months of refractory cutaneous LP [35]. In addition, Böll et al. reported the effective use of abrocitinib or upadacitinib in seven patients (six women and one man) with refractory cutaneous LP [36]. Interestingly, two patients showed complete relief from pruritus within the first days of therapy and all patients had >four points reduction in numeric rating scale (NRS) pruritus by day 21 [36]. Adverse events typically related to JAKI, like infections, venous thromboembolisms, and major cardiovascular events, were not reported [36]. Moreover, Ball et al. successfully treated a female patient with extensive cutaneous LP using oral deucravacitinib 6 mg once daily, leading to a reduction of body surface area (BSA) involvement from 80% to 20% within two months [37]. The effectiveness of oral tofacitinib in recalcitrant hypertrophic LP was confirmed in a retrospective study on 15 patients [38]. According to the authors, pruritus resolved within a mean of 8.6 days and the mean time to achieve resolution of lesions was 4.7 weeks [38]. However, two patients did not show any improvement, and six patients developed side effects, like dyslipidemia, upper respiratory infection, fever and folliculitis [38]. We summarized the most important details in Table 1 for practical purposes.

## 7. Use of JAK Inhibitors for Oral Lichen Planus

The effectiveness and safety of JAKIs for treating OLP has been extensively reported [39,40]. Upadacitinib has been used as off-label therapy in three different case reports. Kooybaran et al. firstly reported the case of a 59-year-old woman with a five-year history of painful erosive OLP who showed a massive improvement after four weeks on upadacitinib 15 mg daily [41]. Furthermore, Balestri et al. reported the case of a 45-year-old woman with a three-week history of erosive OLP successfully treated with upadacitinib, 15 mg daily [42]. Baricitinib 3.4 mg twice daily has been reported as successful in a 63-year-old Caucasian woman with chronic alopecia areata and coincidental non-erosive OLP [43]. In addition, a female patient with recalcitrant erosive OLP was successfully treated with a combination of upadacitinib 15 mg daily and methotrexate [44]. Moreover, Slater et al. described a 70% improvement within one month on upadacitinib in one patient with non-erosive OLP [45]. Stolte et al. reported that deucravacitinib 6 mg daily had a beneficial effect in three patients with OLP [46]. Although monotherapy with deucravacitinib did not lead to complete resolution of the chronic lesions after 12 weeks, all three patients showed a good clinical response with an improvement of reticular lesions and a reduction in the size of the erosions [47]. Gowda et al. reported a drastic improvement after one month and an almost complete resolution after three months on tofacitinib 5 mg twice daily in three patients with erosive OLP [46]. Moreover, tofacitinib has been reported as effective in another case series with six patients with erosive OLP [48]. We summarized the most important details in Table 2 for practical purposes.

## 8. Use of JAK Inhibitors for Lichen Planopilaris

The efficacy of JAKIs has been reported in several papers. A total of eight retrospective studies involving 172 patients have evaluated the use of tofacitinib (oral/topical), baricitinib (oral), and ruxolitinib (topical) (Table 3) [49,50,51,52,53,54,55,56]. A recent systematic review suggested that JAKI may be a valuable option for refractory LPP/FFA patients, with few reported adverse effects [57]. It has been reported that oral tofacitinib demonstrated slightly greater effectiveness than baricitinib, probably due to its stronger inhibition of IFN-γ, while topical tofacitinib and ruxolitinib also showed positive responses [57]. Additionally, a randomized, placebo-controlled phase 2a trial investigating brepocitinib, a tyrosine kinase 2/Janus kinase 1 inhibitor (45 mg/day), in 37 patients with FFA, LPP, or centrifugal cicatricial alopecia showed a significant reduction in clinical severity scores across all cicatricial alopecia subtypes through week 48 [58].

## 9. Use of JAKIs for Nail Lichen Planus

Current data are limited to case reports, and no clinical trials of JAKIs for NLP are planned. To date, five case reports have documented the use of JAKIs for NLP (Table 4) [59,60,61,62]. These reports suggest that JAKIs have promising efficacy and safety in the treatment of NLP.

## 10. Conclusions

LP is a common inflammatory disease characterized by different clinical features. Indeed, LP can affect skin, mucosae and appendages. The heterogeneity of its presentation is paralleled by the variability in treatment response, with many cases proving refractory to conventional therapies. Although corticosteroids and immunosuppressants remain the mainstay of treatment, their long-term use is often limited by safety concerns and variable efficacy. In recent years, JAKIs dramatically changed the therapeutic management of several inflammatory skin diseases, providing effective and safe therapy for alopecia areata, atopic dermatitis and psoriasis. Although JAKIs are still an off-label therapy for LP, emerging clinical evidence indicates that most of the patients showed an improvement of the clinical picture and disease prognosis on JAKIs, while only a small minority complained of adverse events. The growing body of data supporting a key pathogenic role for the JAK/STAT pathway in LP provides a strong biological rationale for their use. However, the current evidence is mostly derived from case reports and small case series. Larger, controlled clinical trials are warranted to better define their role, establish optimal dosing regimens, and assess long-term safety in this setting.

## Figures and Tables

**Table 1:** Summary of papers reporting the use of JAK inhibitors for cutaneous lichen planus.

Author	Year	Study Type	JAK Inhibitor	Result
Seiringer et al. [33]	2020	Case report	Oral tofacitinib	Reduction of pruritus on a NRS scale from 10/10 (baseline) to 0/10 (week 20)
Youssef et al. [34]	2023	Case report	Oral tofacitinib	Resolution of pruritus and improvement of patches at week 4
Zundell et al. [35]	2023	Case report	Oral upadacitinib	Successfully treated
Böll et al. [36]	2024	Case series	Oral abrocitinib or upadacitinib	Two patients obtained complete relief from pruritus within the first days; 7 out of 7 patients had >4 points reduction in NRS pruritus by day 21
Ball et al. [37]	2024	Case report	Oral deucravacitinib	Reduction of BSA involvement from 80% to 20% at week 8
Sharath et al. [38]	2025	Case series	Oral tofacitinib	Pruritus resolved in a mean of 8.6 days; mean time to achieve resolution of lesions was 4.7 weeks

Legend: body surface area (BSA); numerical rating scale (NRS).

**Table 2:** Summary of papers reporting the use of JAK inhibitors for oral lichen planus.

Author	Year	Study Type	JAK Inhibitor	Result
Kooybaran et al. [41]	2021	Case report	Oral upadacitinib	Pain at baseline 7/10 on NRS; pain at week three 5/10 on NRS
Balestri et al. [42]	2022	Case report	Oral upadacitinib	Complete healing of the oral lesions after 7 days
Moussa et al. [43]	2022	Case report	Oral baricitinib	Near complete resolution of the oral irritation and discomfort at week 16
Landells et al. [44]	2023	Case report	Oral upadacitinib	Almost complete clearance of oral lesions
Slater et al. [45]	2024	Case report	Oral upadacitinib	A 70% improvement within 4 weeks
Stolte et al. [46]	2024	Case series	Oral deucravacitinib	Improvement of oral lesions at week 12
Gowda et al. [47]	2024	Case series	Oral tofacitinib	Reduction in ODSS from the baseline was 70% at week 12
Mansouri et al. [48]	2024	Case series	Oral tofacitinib	The mean pain alleviation score was 9.16 on the VAS; symptom improvement began 1.33 months after starting treatment (mean value)

Legend: numerical rating scale (NRS); oral disease severity score (ODSS); visual analog scale (VAS).

**Table 3:** Summary of studies with JAK inhibitors for lichen planopilaris.

Author	Year	Study Type	JAK Inhibitor	Result
Yang et al. [49]	2018	Retrospective	Oral tofacitinib	Improvement in 8 out of 10 patients; LPPAI before and after treatment was measured in 7 patients (6.22 before treatment, 3.08 after treatment)
Plante et al. [50]	2020	Retrospective	Oral tofacitinib and topical tofacitinib	Improvement in 2 out of 3 patients on topical therapy and in 5 out of 6 patients on oral therapy
Moussa et al. [51]	2022	Retrospective	Oral baricitinib	Five of the 7 patients with LPP demonstrated an initial reduction in the median LPPAI score of 2.8 (46.5%); 3 of the 5 patients with FFA demonstrated an initial reduction in the median LPPAI score of 5.6 (83.8%)
Dunn et al. [52]	2023	Case series	Topical ruxolitinib 1.5% or oral baricitinib	Case 1 (topical ruxolitinib 1.5%): Initial LPPAI of 7; at week 12, LPPAI score of 2
				Case 2 (topical ruxolitinib 1.5%): Initial LPPA of 8; at week 15, LPPAI of 3
				Case 3 (oral baricitinib): Initial LPPAI of 7; at week 4, LPPAI of 2
Chen et al. [53]	2024	Retrospective	Topical tofacitinib	Out of 38 patients, 92.1% showed an improvement
Gonzalez Matheus et al. [54]	2024	Case series	Oral tofacitinib and oral baricitinib	Clinical hair regrowth in 3 patients, reduced patch size in 1 patient and reduced itching and burning symptoms in 1 patient
Goodarzi et al. [55]	2025	Retrospective	Oral tofacitinib	Nine (12.2%) patients did not respond; 16 (21.6%) patients responded within 1–3 months; 18 (24.3%) patients responded within 3–6 months; 25 (33.8%) patients responded within 6–12 months; 6 (8.1%) patients responded within 12–24 months.
Williams et al. [56]	2025	Retrospective	Topical ruxolitinib	The average percentage reduction in LPPAI score was 34%, with 7 patients demonstrating a <25% reduction, 9 demonstrating a 25% to 75% reduction, and 4 demonstrating a >75% reduction.

Legend: frontal fibrosing alopecia (FFA); lichen planopilaris (LPP); lichen planopilaris activity index (LPPAI).

**Table 4:** Summary of case reports reporting the use of JAK inhibitors for nail lichen planus.

Author	Year	Study Type	Number of Patients	JAK Inhibitor	Result
Iorizzo et al. [59]	2021	Case report	1	Tofacitinib 5 mg twice per day	Significant improvement in 6 months
Pünchera et al. [60]	2022	Case report	1	Baricitinib 4 mg	Complete remission in 6 months
Huang et al. [61]	2023	Case report	1	Tofacitinib 5 mg twice per day	Significant improvement in 6 months
He et al. [62]	2023	Case report	1	Baricitinib 4 mg	Complete remission in 6 months
He et al. [63]	2024	Case report	1	Abrocitinib 100 mg	Significant improvement in 6 months

## Data Availability

No new data were created or analyzed in this study.

## References

[1] Solimani F., Forchhammer S., Schloegl A., Ghoreschi K., Meier K. (2021). Lichen planus—A clinical guide. J. Dtsch. Dermatol. Ges..

[2] Le Cleach L., Chosidow O. (2012). Clinical practice. Lichen planus. N. Engl. J. Med..

[3] Li C., Tang X., Zheng X., Ge S., Wen H., Lin X., Chen Z., Lu L. (2020). Global Prevalence and Incidence Estimates of Oral Lichen Planus: A Systematic Review and Meta-analysis. JAMA Dermatol..

[4] Vičić M., Hlača N., Kaštelan M., Brajac I., Sotošek V., Prpić Massari L. (2023). Comprehensive Insight into Lichen Planus Immunopathogenesis. Int. J. Mol. Sci..

[5] Solimani F., Ghoreschi K. (2024). Januskinaseinhibitoren für dermatologische Erkrankungen. Dermatologie.

[6] Miot H.A., Criado P.R., de Castro C.C.S., Ianhez M., Talhari C., Ramos P.M. (2023). JAK-STAT pathway inhibitors in dermatology. An. Bras. Dermatol..

[7] Steffen C., Dupree M.L. (2004). Louis-Frédéric Wickham and the Wickham’s striae of lichen planus. Skinmed.

[8] Wagner G., Rose C., Sachse M.M. (2013). Clinical variants of lichen planus. J. Dtsch. Dermatol. Ges..

[9] Whittington C.P., Saleh J.S., Bresler S.C., Patel R.M. (2024). Hypertrophic Lichen Planus: An Up-to-Date Review and Differential Diagnosis. Arch. Pathol. Lab. Med..

[10] Manz B., Paasch U., Sticherling M. (2005). Squamous cell carcinoma as a complication of long-standing hypertrophic lichen planus. Int. J. Dermatol..

[11] Tonsager M., Crutchfield C.E. (2004). Atrophic lichen planus. Dermatol. Nurs..

[12] Robles-Méndez J.C., Rizo-Frías P., Herz-Ruelas M.E., Pandya A.G., Ocampo Candiani J. (2018). Lichen planus pigmentosus and its variants: Review and update. Int. J. Dermatol..

[13] Kanzaki T., Otake N., Nagai M. (1992). Eruptive lichen planus. J. Dermatol..

[14] Didona D., Caposiena Caro R.D., Sequeira Santos A.M., Solimani F., Hertl M. (2022). Therapeutic strategies for oral lichen planus: State of the art and new insights. Front. Med..

[15] Didona D., Hinterseher J., Eming R. (2022). Bullöse Autoimmundermatosen der Schleimhaut. Dermatologie.

[16] Didona D., Hertl M. (2022). Detection of anti-desmoglein antibodies in oral lichen planus: What do we know so far. Front. Immunol..

[17] Didona D., Schmidt M.F., Meier K., Mesas-Fernandez A., Maglie R., Antiga E., Klemp M., Yazdi A.S., Ghoreschi K., Hertl M. (2024). Pathogenic relevance of antibodies against desmoglein 3 in patients with oral lichen planus. J. Dtsch. Dermatol. Ges..

[18] Fitzpatrick S.G., Hirsch S.A., Gordon S.C. (2014). The malignant transformation of oral lichen planus and oral lichenoid lesions: A systematic review. J. Am. Dent. Assoc..

[19] Georgakopoulou E.A., Troupis T.G., Troupis G., Gorgoulis V.G. (2011). Update of the cancer-associated molecular mechanisms in oral lichen planus, a disease with possible premalignant nature. J. BUON.

[20] Kerkemeyer K.L.S., Eisman S., Bhoyrul B., Pinczewski J., Sinclair R.D. (2021). Frontal fibrosing alopecia. Clin. Dermatol..

[21] Wagner G., Meyer V., Sachse M.M. (2016). Frontal fibrosierende Alopezie Kossard. Hautarzt.

[22] Verma G. (2019). Graham-Little-Piccardi-Lasseur Syndrome. Indian Dermatol. Online J..

[23] Gupta M.K., Lipner S.R. (2021). Review of Nail Lichen Planus: Epidemiology, Pathogenesis, Diagnosis, and Treatment. Dermatol. Clin..

[24] Hwang J.K., Grover C., Iorizzo M., Lebwohl M.G., Piraccini B.M., Rigopoulos D.G., Lipner S.R. (2024). Nail psoriasis and nail lichen planus: Updates on diagnosis and management. J. Am. Acad. Dermatol..

[25] McLornan D.P., Pope J.E., Gotlib J., Harrison C.N. (2021). Current and future status of JAK inhibitors. Lancet.

[26] Ghoreschi K., Laurence A., O’Shea J.J. (2009). Selectivity and therapeutic inhibition of kinases: To be or not to be?. Nat. Immunol..

[27] O’Shea J.J., Schwartz D.M., Villarino A.V., Gadina M., McInnes I.B., Laurence A. (2015). The JAK-STAT pathway: Impact on human disease and therapeutic intervention. Annu. Rev. Med..

[28] Pietschke K., Holstein J., Meier K., Schäfer I., Müller-Hermelink E., Gonzalez-Menendez I., Quintanilla-Martinez L., Ghoreschi F.C., Solimani F., Ghoreschi K. (2021). The inflammation in cutaneous lichen planus is dominated by IFN-ϒ and IL-21-A basis for therapeutic JAK1 inhibition. Exp. Dermatol..

[29] Shao S., Tsoi L.C., Sarkar M.K., Xing X., Xue K., Uppala R., Berthier C.C., Zeng C., Patrick M., Billi A.C. (2019). IFN-γ enhances cell-mediated cytotoxicity against keratinocytes via JAK2/STAT1 in lichen planus. Sci. Transl. Med..

[30] Schmidt T., Solimani F., Pollmann R., Stein R., Schmidt A., Stulberg I., Kühn K., Eming R., Eubel V., Kind P. (2018). TH1/TH17 cell recognition of desmoglein 3 and bullous pemphigoid antigen 180 in patients with lichen planus. J. Allergy Clin. Immunol..

[31] Tian Y., Zajac A.J. (2016). IL-21 and T Cell Differentiation: Consider the Context. Trends Immunol..

[32] Motamed-Sanaye A., Khazaee Y.F., Shokrgozar M., Alishahi M., Ahramiyanpour N., Amani M. (2022). JAK inhibitors in lichen planus: A review of pathogenesis and treatments. J. Dermatol. Treat..

[33] Seiringer P., Lauffer F., Pilz A.C., Boehmer D., Biedermann T., Eyerich K. (2020). Tofacitinib in Hypertrophic Lichen Planus. Acta Derm. Venereol..

[34] Youssef S., Bordone L.A. (2023). Oral tofacitinib effectively treating eruptive and hypertrophic cutaneous lichen planus. JAAD Case Rep..

[35] Zundell M.P., Kaminetsky J., Lebwohl M., Gottlieb A.B. (2023). Successful Treatment of Lichen Planus with Oral Upadacitinib. J. Drugs Dermatol..

[36] Böll S.L., Zahn C.A., Schlapbach C. (2024). Rapid and sustained improvement of cutaneous lichen planus with oral JAK1 inhibitors. J. Eur. Acad. Dermatol. Venereol..

[37] Ball G.D., Golant A. (2024). A Case of Extensive Lichen Planus Treated with Deucravacitinib. Cureus.

[38] Sharath S., Sardana K., Khurana A., Yadav A., Singh A. (2025). Therapy resistant hypertrophic lichen planus and its response to oral tofacitinib with a priori tissue cytokine expression: A real-world hospital-based study. Arch. Dermatol. Res..

[39] Popa C., Sciuca A.M., Onofrei B.-A., Toader S., Condurache Hritcu O.M., Boțoc Colac C., Porumb Andrese E., Brănișteanu D.E., Toader M.P. (2024). Integrative Approaches for the Diagnosis and Management of Erosive Oral Lichen Planus. Diagnostics.

[40] Wu T., Bai Y., Jing Y., Chen F. (2024). What can we learn from treatments of oral lichen planus?. Front. Cell. Infect. Microbiol..

[41] Kooybaran N.R., Petzold G., Ströbel P., Schön M.P., Mössner R. (2021). Alleviation of erosive oral and esophageal lichen planus by the JAK1 inhibitor upadacitinib. J. Dtsch. Dermatol. Ges..

[42] Balestri R., Bortolotti R., Rech G., Girardelli C.R., Zorzi M.G., Magnano M. (2022). Treatment of Oral Erosive Lichen Planus with Upadacitinib. JAMA Dermatol..

[43] Moussa A., Colla T., Morrison B., Sinclair R. (2022). Effective treatment of oral lichen planus with the JAK inhibitor baricitinib. Australas. J. Dermatol..

[44] Landells F.M., Goudie S., McGrath J., Tibbo J., Landells I. (2023). Successful treatment of erosive lichen planus with Upadacitinib complicated by oral squamous cell carcinoma. SAGE Open Med. Case Rep..

[45] Slater K., Halash K., Kartono F. (2024). Oral Lichen Planus Successfully Treated With Upadacitinib. J. Drugs Dermatol..

[46] Stolte K.N., Mesas-Fernández A., Meier K., Klein E.K., Dommisch H., Ghoreschi K., Solimani F. (2024). TYK2 inhibition with deucravacitinib ameliorates erosive oral lichen planus. Exp. Dermatol..

[47] Gowda S.K., Thakur V., Behera B., Garg S., Sahu D.K., Sethy M., Ayyanar P. (2024). The efficacy and safety of oral tofacitinib in oral erosive lichen planus: A case series. Int. J. Dermatol..

[48] Mansouri P., Jafari M.A., Chalangari R., Roohaninasab M., Goodarzi A. (2024). Successful Treatment of Erosive Lichen Planus With Tofacitinib: A Case Series and Review of the Literature. Clin. Med. Insights Case Rep..

[49] Yang C.C., Khanna T., Sallee B., Christiano A.M., Bordone L.A. (2018). Tofacitinib for the treatment of lichen planopilaris: A case series. Dermatol. Ther..

[50] Plante J., Eason C., Snyder A., Elston D. (2020). Tofacitinib in the treatment of lichen planopilaris: A retrospective review. J. Am. Acad. Dermatol..

[51] Moussa A., Bhoyrul B., Asfour L., Kazmi A., Eisman S., Sinclair R.D. (2022). Treatment of lichen planopilaris with baricitinib: A retrospective study. J. Am. Acad. Dermatol..

[52] Dunn C., Griffith V., Coican A., Dane A., Chow W., Aneja S., Nathoo R., Leavitt A., Hawkins S.D. (2023). Janus kinase inhibition for the treatment of refractory frontal fibrosing alopecia: A case series and review of the literature. JAAD Case Rep..

[53] Chen L.-C., Ogbutor C., Kelley K.J., Pickford J.R., Senna M.M. (2024). Prevalence of lichen planopilaris and frontal fibrosing alopecia in the United States: An All of Us database analysis. Int. J. Dermatol..

[54] Gonzalez Matheus G.A., Khosrotehrani K. (2024). Treatment of lichen planopilaris with Janus kinase inhibitors. Australas. J. Dermatol..

[55] Goodarzi M., Dadkhahfar S., Yahyaei F., Razzaghi Z., Moravvej H. (2025). Efficacy and Safety of Tofacitinib in Lichen Planopilaris: A Retrospective Series of 74 Patients. Arch. Iran. Med..

[56] Williams K.N., Perez S.M., Burroway B., Tosti A. (2025). Topical ruxolitinib in the management of frontal fibrosing alopecia and/or lichen planopilaris: A single-center retrospective cohort study. J. Am. Acad. Dermatol..

[57] Wang C., Jiang Y. (2025). Efficacy and safety of JAK inhibitors in treating lichen planopilaris or frontal fibrosing alopecia. J. Eur. Acad. Dermatol. Venereol..

[58] David E., Shokrian N., Del Duca E., Meariman M., Dubin C., Hawkins K., Andrews E., Sikand S., Singer G., Oemar B. (2025). A phase 2a trial of brepocitinib for cicatricial alopecia. J. Am. Acad. Dermatol..

[59] Iorizzo M., Haneke E. (2021). Tofacitinib as Treatment for Nail Lichen Planus Associated with Alopecia Universalis. JAMA Dermatol..

[60] Pünchera J., Laffitte E. (2022). Treatment of Severe Nail Lichen Planus with Baricitinib. JAMA Dermatol..

[61] Huang J., Shi W. (2023). Successful treatment of nail lichen planus with tofacitinib: A case report and review of the literature. Front. Med..

[62] He J., Weng T., Zhu W., Yang Y., Li C. (2023). Alleviation of isolated nail lichen planus by the JAK1/2 inhibitor Baricitinib: A case report. J. Dermatol. Treat..

[63] He J., Yang Y. (2024). Janus kinase 1 inhibitor abrocitinib for isolated nail lichen planus: A case report and literature review. J. Dermatol. Treat..

